# Identifying immune burden status and predictors via the non-classical type 2 inflammatory axis in allergic rhinitis

**DOI:** 10.3389/fimmu.2026.1936486

**Published:** 2026-09-30

**Authors:** Yi He, Beirong Liu, Xuegui Liu, Xinman Hu, Qing Liu, Zhuoping Liang

**Affiliations:** Department of Otolaryngology Head and Neck Surgery, the Affiliated Hospital of Southwest Medical University, Luzhou, China

**Keywords:** allergic rhinitis, biomarker, clinical response, immunity, local immune signature

## Abstract

**Background:**

While the roles of classical type 2 (T2) inflammatory factors in allergic rhinitis (AR) have been well characterized, the roles of non-classical T2 inflammation factors are less explored. This study aimed to elucidate the roles and predictive significance of non-classical T2 inflammatory factors in AR.

**Methods:**

AR-related datasets were retrieved from GEO for bioinformatics analysis. A total of 64 AR patients and 42 healthy controls were included. Non-classical T2 factors and T1 cytokines were measured by ELISAs. The SNOT-22 and VAS scores were used to evaluate the disease severity of AR. PCA-PAM clustering, MCA and correlation network analysis were performed to explore the inflammatory pattern and interactions of non-classical T2 factors, respectively. ROC curves were constructed to evaluate the predictive performance. All biomarkers were robustly standardized to healthy control medians and IQRs prior to PCA, MCA, and ROC analyses. Internal validation was performed via LOOCV, bootstrap resampling, and 10-fold cross-validation.

**Results:**

Serum ALOX15 and ratios of ALOX15, CLC, CST1, POSTN, TSLP, and IL-33 to T1 cytokines were significantly elevated in AR patients, with concordant upregulation of ALOX15 at transcript and protein levels in both nasal mucosa and serum. PAM clustering of PCA-derived T1/T2 biomarkers stratified AR patients into the low-immune-response type (LIR-AR, n = 40) and the high-immune-response type (HIR-AR, n = 24) endotypes. MCA identified CST1, POSTN, and CLC as the top discriminators, with IL-33 spatially separated from downstream effectors. CST1 levels and the ALOX15/IFN-γ ratios were correlated with the VAS scores for rhinorrhea and nasal itching, respectively. The ALOX15/TNF-α ratio exhibited good predictive accuracy for AR (AUC = 0.856 ± 0.106).

**Conclusions:**

AR was characterized by T1/T2 imbalance, with elevated ALOX15 and increased T2/T1 ratios in both nasal mucosa and serum, suggesting ALOX15 as a potential biomarker. CST1, POSTN, and CLC were core variables distinguishing LIR-AR from HIR-AR and may be useful for immunotype differentiation. CST1 expression and the ALOX15/IFN-γ ratio correlated with rhinorrhea and nasal itching, respectively; however, these correlation-based findings require cautious interpretation regarding causality. The ALOX15/TNF-α ratio showed good diagnostic value for AR, with these exploratory findings requiring validation in larger independent cohorts and functional studies.

## 1 Introduction

Allergic rhinitis (AR) imposes a significant global health burden (1), with a prevalence ranging from 5% to 50% (2). AR is a chronic immunoglobulin (Ig)E-mediated inflammatory disorder of the nasal mucosa that is triggered by environmental allergens and is clinically characterized by nasal itching, sneezing, rhinorrhea, and congestion (3). The core pathophysiology of AR involves a type 2 (T2) immune response driven by T2 cell-derived inflammatory factors that initiate an IgE/mast cell/eosinophil cascade (4). The roles of classical T2 factors (e.g., interleukin (IL)-4, IL-5, and IL-13) in the pathogenesis of AR are well established as the core of T2 immunity (5). These factors interact with the T1 core effectors interferon-gamma (IFN-γ) and tumor necrosis factor-alpha (TNF-α), thereby promoting the Th1/Th2 immune imbalance (6).

With the development of high-throughput technology, a series of non-classical T2 inflammatory markers produced by epithelial cells and immune cells have been confirmed to be widely involved in the inflammatory response to allergic diseases. For example, the lipid metabolic enzyme arachidonate 15-lipoxygenase (ALOX15) is closely related to the progression of airway inflammation (7); eosinophil (EOS)-associated inflammatory mediators, including eotaxin, Charcot–Leyden crystal (CLC) and eosinophil cationic protein (ECP), can reflect the extent of EOS infiltration (8). Periostin (POSTN) and cystatin SN (CST1) play important roles in regulating the AR microenvironment (9, 10). Alarmins released by damaged epithelial cells, including IL-33 and thymic stromal lymphopoietin (TSLP), have been identified as bridges linking environmental exposure to the initiation of T2 immunity (11). These non-classical T2 inflammatory factors have greatly enriched our understanding of the inflammatory mechanisms of AR.

This study aimed to screen non-classical T2 factors that may play vital roles in AR by analyzing public databases. Enzyme-linked immunosorbent assays (ELISAs) were used to detect the serum levels of non-classical T2 factors and classical T1 cytokines in AR patients and healthy controls, followed by a comparative analysis of the results. Principal component analysis (PCA)-assisted partitioning around medoids (PAM) clustering and multiple correspondence analysis (MCA) were applied to analyze and visualize the overall correlation patterns between serum levels of T2 factors and T1 cytokines in patients with AR. An analysis of the correlation network was subsequently performed to explore the interactions among non-classical T2 factors in AR patients. The correlations of non-classical T2 inflammatory factors and their ratios to T1 cytokines with the scores measuring the severity of AR were analyzed. Finally, receiver operating characteristic (ROC) curves were constructed to evaluate the efficacy of promising serum inflammatory factor ratios for predicting AR. These approaches can provide new insights into the inflammatory patterns of AR and support biomarker-based diagnostic strategies.

## 2 Methods

### 2.1 Bioinformatics analysis of the microarray data

Candidate non-classical T2 inflammatory factors were identified by analyzing datasets from the Gene Expression Omnibus (GEO) database (https://www.ncbi.nlm.nih.gov/geo/). The inclusion criteria were as follows: (1) human datasets with ≥ 10 samples; (2) at least 5 patients and 5 controls; and (3) the availability of raw or processed expression data from microarray experiments. Normalized gene expression matrices were processed using R software (v4.5.1) and the limma package (v3.58.1). Non-classical T2 inflammatory factors (ALOX15, CLC, CST1, ECP, eotaxin, POSTN, TSLP, and IL-33) and classical T1 inflammatory factors (IFN-γ and TNF-α) were the primary focus of the analysis. After quality control and background correction, a differential expression analysis was conducted with an adjusted *P* value (false discovery rate (FDR) -corrected) < 0.05 and a |log2 fold change (log2FC) | > 1 set as the significance thresholds. PCA was performed to visualize intergroup separation.

Given the relatively limited sample size of the discovery cohort (GSE43523 dataset), we adopted a stepwise analytical framework. The GSE43523 dataset was designated as a preliminary exploratory screening set rather than a confirmatory cohort, serving primarily to narrow the candidate gene pool and generate hypotheses. We performed leave-one-out cross-validation (LOOCV) and bootstrap resampling (n = 1000 iterations) on the pre-selected panel of inflammatory factor genes to evaluate the internal robustness of the results derived from this screening set. Genes were considered stable if they exhibited a coefficient of variation (CV) < 0.5 and a 95% confidence interval for log2FC that excluded zero. Subsequently, based on predefined criteria, including the nasal mucosal tissue origin, clear clinical phenotyping, adequate sample size, and complete expression data for the candidate genes, we pursued external validation in an independent AR cohort (GSE101720).

### 2.2 Subjects

A total of 64 patients with AR and 42 healthy control subjects from the Affiliated Hospital of Southwest Medical University were enrolled. All patients or their legal guardians (for patients under 18 years of age) provided written informed consent prior to enrollment. The diagnosis of AR was based on the ICAR-Allergic Rhinitis 2023 (2) and Allergic Rhinitis and its Impact on Asthma (ARIA) Phase 4 (2018) (12). The inclusion criteria for AR patients were as follows: (1) diagnosed with AR; (2) agreed to participate in this study; (3) aged ≥ 5 years; and (4) had complete clinical data. The exclusion criteria were as follows: (1) patients diagnosed with concurrent immune system diseases or recent infections; (2) patients who were complicated with nasal polyps, severe nasal septum deviation, sinusitis, atrophic rhinitis, or other nasal diseases; and (3) patients who used topical or systemic glucocorticoids, antihistamines, or immunotherapy within 4 weeks prior to serum sample collection. This study was approved by the Ethics Committee of the Affiliated Hospital of Southwest Medical University (No. KY2026254).

### 2.3 Detection of the serum levels of non-classical T2 inflammatory factors and T1 inflammatory factors using ELISAs

Serum levels of ALOX15, CLC, CST1, ECP, eotaxin, POSTN, TSLP, IL-33, IFN-γ, and TNF-α were quantified using commercial ELISA kits (YOBIBO Trading Co., Ltd., Shanghai, China) according to the manufacturer’s instructions. Briefly, samples and serially diluted standards were applied to precoated plates. After an incubation with a biotinylated detection antibody and streptavidin-HRP, the reaction was developed with TMB substrate. The optical density was measured at 450 nm, and the sample concentrations of the samples were interpolated from the standard curve and corrected for the dilution factor.

### 2.4 PAM clustering analysis for immune endotyping based on PCA-derived scores

Each biomarker was first standardized using the median and interquartile range (IQR) of the healthy control group to eliminate dimension scale effects and increase the stability of the model. PCA was subsequently performed separately on the T1 pathway marker panel (IFN-γ and TNF-α) and the T2 pathway marker panel (ALOX15, CLC, CST1, ECP, eotaxin, POSTN, TSLP, and IL-33), and the first principal component (PC) of each panel was extracted as the T1 composite score and T2 composite score, respectively.

Then, unsupervised clustering of patients was performed using the PAM algorithm. The optimal number of clusters was determined by calculating the silhouette coefficient. Furthermore, bootstrap resampling with 1000 iterations was employed to validate the stability of the PAM clustering results.

### 2.5 Visualization of immunoreactive endotypes using MCA

MCA was further employed as a tool in this study to visually display the association patterns between the immune subtypes identified by PAM clustering and the expression patterns of key inflammatory factors. The input variables for MCA included the T1 and T2 pathway composite scores, as well as the median-dichotomized categorical variables of inflammatory factor expression levels in AR patients, all of which were converted into categorical variables and incorporated as active variables. The cluster labels generated by PAM clustering were introduced as supplementary qualitative variables, which did not participate in dimension construction but were used solely for *post hoc* visualization and projection. Notably, MCA did not serve as a primary classification method in this study; rather, it was positioned as a visualization and auxiliary interpretation tool for the PAM clustering results. The core basis for evaluating clustering validity was the silhouette coefficient and bootstrap resampling (1000 iterations) results, and the explained variance rates of the MCA were not considered criteria for assessing classification validity.

### 2.6 Interaction networks of T2 inflammatory factors in AR patients

Interaction networks were further explored by constructing correlation network plots, with correlations assessed using Pearson’s or Spearman’s tests based on the data distribution. A two-sided *P* value < 0.05 was considered to indicate statistical significance.

### 2.7 Construction of ROC curves to evaluate the predictive performance

Logistic regression analysis was employed to construct diagnostic models, and ROC curves were plotted to evaluate the predictive performance before and after age adjustment. Stratified 10-fold cross-validation repeated 10 times was further performed for internal validation. With a fixed random seed, all the samples were shuffled and divided into ten nonoverlapping subgroups. Stratified sampling was adopted to maintain a consistent patient–control ratio within each fold. In each iteration, nine folds were used for model training, and the remaining fold was used for validation. After ten iterative cycles, the mean area under the curve (AUC) of the ten validation folds was calculated to quantify the predictive performance. The differences between the training and validation AUCs across folds were examined to assess potential overfitting. The AUC thresholds for discriminative performance were set as follows: poor (< 0.7), moderate (0.7 – 0.8), good (0.8 – 0.9), and excellent (≥ 0.9). The optimal cutoff was determined by maximizing Youden’s index (J = Sensitivity + Specificity − 1) and corresponding sensitivity and specificity were reported.

### 2.8 Visual analog scale score

The VAS score was used to evaluate nasal itching, sneezing, rhinorrhea, and nasal obstruction in AR patients, with total scores summed according to our established protocol (13).

### 2.9 Twenty-two-item sinonasal outcome test score

The SNOT-22 scoring method, comprising twenty-two questions each scored from 0 to 5 points (total score of 0–110 points), was used to assess the impact of AR on patients’ physiological problems, functional limitations, and emotional problems within one week. The specific scoring protocol followed that described in a previous study by our research group (13).

### 2.10 Statistical analysis

R software (Version R4.5.1; R Foundation for Statistical Computing, Vienna, Austria), including the packages readxl, ggplot2, pROC, caret, FactoMineR, cluster and dplyr, and IBM SPSS Statistics 27 (Version 27; IBM Corp., Armonk, NY, USA) were used to analyze the data. Normally distributed continuous variables were presented as means ± standard deviations and were compared using the *t* test, whereas nonnormally distributed data were presented as medians (IQRs) and were compared using the Mann–Whitney *U* test. As the age distribution differed between the AR and control groups, group differences were adjusted for age using analysis of covariance (ANCOVA) for normally distributed variables and a generalized linear model (GLM) (gamma distribution, log link) for nonnormally distributed variables. LOOCV and bootstrap resampling (n = 1000 iterations) were employed to evaluate the internal stability of the GSE43523 dataset. Genes were considered stable if they exhibited a CV < 0.5 and a 95% confidence interval for log2FC that excluded zero. Unsupervised PAM clustering was performed using PCA-derived T1 and T2 composite scores, with the number of clusters determined by calculating the silhouette coefficient and stability assessed via bootstrap resampling (1000 iterations). MCA was implemented with the FactoMineR package (R.4.5.1), and biplots were produced using the fviz_mca_biplot function. Group differences in sample coordinates on MCA dimensions were assessed with the Mann–Whitney *U* test. A logistic regression analysis was performed to construct diagnostic models, and ROC curves were plotted to evaluate the predictive performance before and after the age adjustment. Internal validation was performed using stratified 10-fold cross-validation repeated 10 times to calculate the mean value of the AUC and assess potential overfitting. A *P* value < 0.05 was considered to indicate statistical significance.

## 3 Results

### 3.1 Identification and validation of non-classical T2 inflammatory signatures in AR patients

We analyzed a nasal mucosal transcriptome dataset (GSE43523) comprising 7 AR patients and 5 healthy controls from the GEO database to identify key non-classical T2 inflammatory markers involved in AR. This study focused on eight T2 markers representing diverse pathophysiological pathways, including ALOX15 (lipid metabolism), CLC and ECP (EOS activation), eotaxin (chemotaxis), TSLP, IL-33, and CST1 (epithelial barrier function), and POSTN (tissue remodeling), alongside the classical T1 cytokines IFN-γ and TNF-α. PCA revealed no clear separation between groups at the whole-genome level (**Figure 1A**). The differential expression analysis revealed the significant upregulation of ALOX15, CLC, and CST1 in AR patients, whereas the expression levels of other targets remained unchanged (**Figure 1B**). The volcano plot confirmed these findings, with the expression of ALOX15 (log2FC = 1.27), CLC (log2FC = 2.57), and CST1 (log2FC = 6.01) showing significant increases (FDR < 0.05; **Figure 1C**). These results suggest that the transcriptional signature of AR in this cohort is characterized by the specific upregulation of ALOX15, CLC, and CST1, highlighting their roles as potential key effectors and markers.

**Figure 1:**
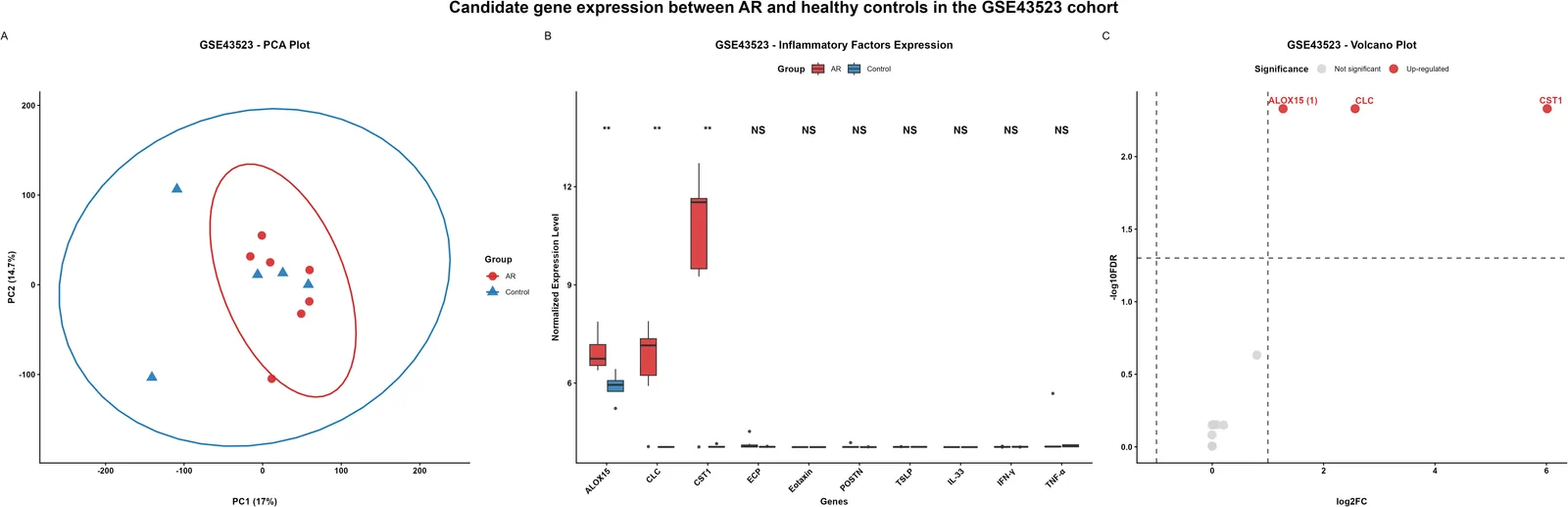
Analysis of the expression of T1 and T2 inflammatory genes in AR patients compared with healthy controls in the GEO database (GSE43523). **(A)** PCA indicated a lack of clear separation between the AR and healthy control groups in terms of gene expression profiles. **(B)** The differential expression analysis revealed that ALOX15, CLC, and CST1 were upregulated in the AR group, and the expression of other T2 inflammatory genes, such as ECP, eotaxin, POSTN, TSLP, and IL-33, and the expression of T1 cytokines, such as IFN-γ and TNF-α, did not differ significantly between the AR group and the control group. **(C)** The volcano plot of the results further showed that ALOX15, CLC and CST1 were significantly highly expressed in the nasal mucosal tissue of AR patients (|log2 FC| > 1 and FDR < 0.05). T, type; AR, allergic rhinitis; GEO, Gene Expression Omnibus; PCA, principal component analysis; ALOX15, arachidonate 15-lipoxygenase; CLC, Charcot–Leyden crystal; CST1, cystatin SN; ECP, eosinophil cationic protein; POSTN, periostin; TSLP, thymic stromal lymphopoietin; IL, interleukin; IFN-γ, interferon-gamma; TNF-α, tumor necrosis factor-alpha; log2FC, log2 fold change; FDR, false discovery rate. ; NS, not significant ; NS, not significant** represents *P* values less than 0.01.

LOOCV and bootstrap resampling (n = 1000 iterations) were applied to assess the stability of the differentially expressed genes (DEGs) and to determine whether these findings were driven by outlier samples. LOOCV demonstrated that the changes in the expression of ALOX15, CST1, and CLC remained stable across all iterations, with the CV consistently below 0.5 and the standard deviation below 1 (**Supplementary Figure 1**). The results of the bootstrap analysis further confirmed the robustness of these upregulated genes, as the 95% confidence intervals of their log2FC values excluded zero (**Supplementary Figure 2**).

We sought to externally validate the results in an independent AR cohort (GSE101720, 17 AR patients vs. 9 controls), which was a publicly available nasal mucosal dataset meeting our predefined inclusion criteria, to evaluate the cross-dataset reproducibility. Although PCA revealed considerable biological heterogeneity inherent to real-world clinical samples, the trends of upregulated ALOX15 and CST1 expression were successfully replicated in this independent cohort (**Figure 2**), supporting the robustness of our findings across datasets.

**Figure 2:**
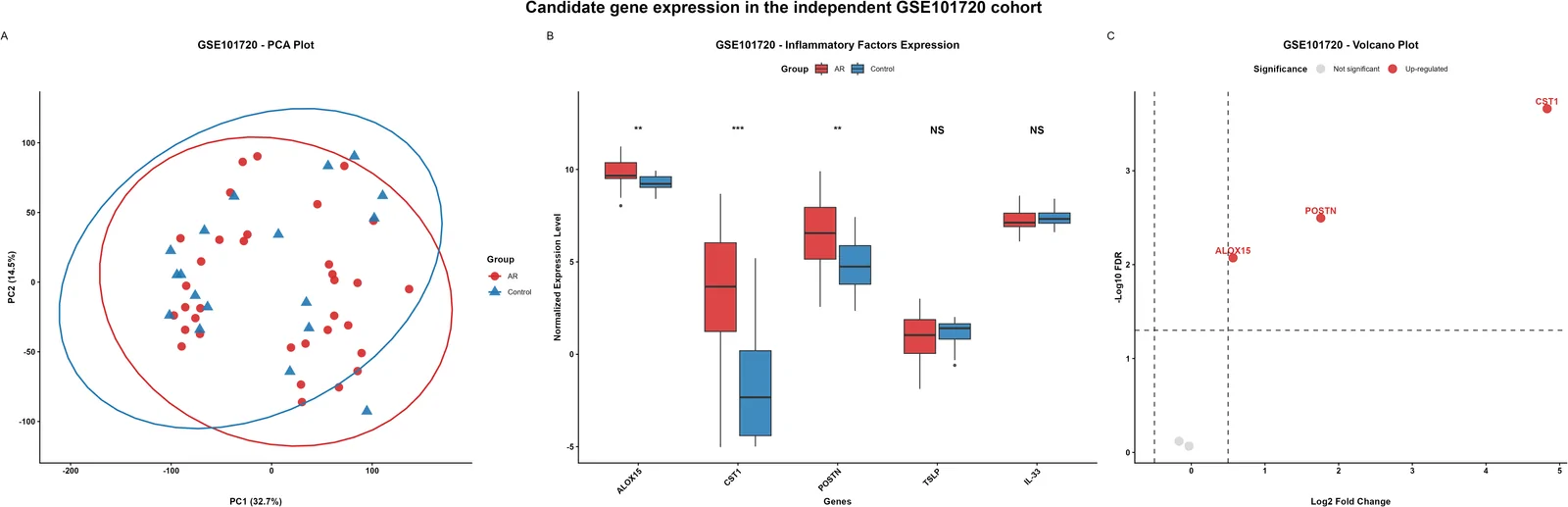
External validation of candidate gene expression in the independent GSE101720 cohort. **(A)** PCA plot showing the overall transcriptional profiles of AR patients (n = 17) and healthy controls (n = 9), revealing substantial biological heterogeneity inherent to real-world clinical samples. **(B)** The differential expression analysis revealed that ALOX15, CST1 and POSTN were upregulated in the AR group but that the expression of other T2 inflammatory genes, such as TSLP and IL-33, did not differ significantly between the AR group and the control group. **(C)** The volcano plot of the results further showed that ALOX15, POSTN and CST1 were significantly highly expressed in the nasal mucosal tissue of AR patients (|log2 FC| > 1 and FDR < 0.05). PCA, principal component analysis; AR, allergic rhinitis; T, type; ALOX15, arachidonate 15-lipoxygenase; CST1, cystatin SN; POSTN, periostin; TSLP, thymic stromal lymphopoietin; IL, interleukin; log2FC, log2 fold change; FDR, false discovery rate; NS, not significant. ** represents *P* values less than 0.01; *** represents *P* values less than 0.001.

### 3.2 Demographic and clinical characteristics of the participants

No significant differences in sex, asthma incidence, or smoking history were detected between the groups (*P* > 0.05). Notably, the AR group was significantly younger (*P* = 0.005), necessitating an adjustment for age in the subsequent analyses. The AR cohort had a total symptom score of 22.89 ± 9.36 and a median SNOT-22 score of 27.50 (IQR: 22.71). The baseline characteristics of the two groups were summarized in **Table 1**.

**Table 1:** Comparison of baseline characteristics between subjects in the AR group and the control group.

Parameters	AR group (n=64)	Control group (n=42)	*P* - value
Gender			
Male, n (%)	35 (54.69)	15 (35.71)	0.056
Female, n (%)	29 (45.31)	27 (64.29)	
Age	18 (19.00)	26 (11.00)	**0.005**
Asthma, n (%)	2 (3.13)	0 (0.00)	0.517
Smoking, n (%)	1 (1.56)	2 (4.76)	0.561
Nasal symptom VAS score			
Obstruction score	5.61 ± 2.67	N/A	–
Rhinorrhea score	6.00 (4.00)	N/A	–
Sneezing score	6.00 (4.00)	N/A	–
Nasal itching score	5.41 ± 2.82	N/A	–
Total nasal symptom score	22.89 ± 9.36	N/A	–
SNOT-22 score	27.50 (22.71)	N/A	–

Bold font indicates *P* < 0.05, indicating a statistically significant difference. AR, allergic rhinitis; n, number; VAS, visual analog scale; N/A, not applicable; SNOT-22, twenty-two-item sino-nasal outcome test.

### 3.3 Serum levels of non-classical T2 inflammatory factors and T1 cytokines in AR patients and healthy controls

The expression of the aforementioned non-classical T2 inflammatory factors and T1 cytokines were verified in serum from AR patients and healthy controls by performing ELISAs. Consistent with the results of the bioinformatics analysis, the level of the non-classical T2 factor ALOX15 was significantly elevated in AR patients (*P* = 0.003). In contrast, the levels of CST1, ECP, eotaxin, and the classical T1 cytokines IFN-γ and TNF-α were markedly decreased in the AR group (all *P* < 0.05). No significant differences were observed in the levels of CLC, POSTN, TSLP, and IL-33 (**Table 2**).

**Table 2:** Comparison of the expression levels of T2 inflammatory factors and T1 cytokines between patients with AR and healthy controls.

Parameters	AR group (n = 64)	Control group (n = 42)	*P* - value	*P* - value (adjusted for age)
T2 inflammatory factors				
ALOX15, (pg/ml)	867.01 ± 418.53	671.43 ± 302.06	**0.010**	**0.003**
CLC, (ng/ml)	40.52 ± 15.68	35.48 ± 10.24	**0.048**	**0.170**
CST1, (ng/ml)	3.40 ± 1.32	3.92 (1.32)	**0.010**	**0.010**
ECP, (pg/ml)	392.25 (409.86)	710.66 ± 279.02	**<0.001**	**<0.001**
Eotaxin, (pg/ml)	346.47 (180.31)	635.67 ± 281.15	**<0.001**	**<0.001**
POSTN, (ng/ml)	4.37 ± 1.43	4.57 ± 1.23	0.464	0.310
TSLP, (pg/ml)	605.64 ± 242.48	643.83 (240.13)	0.120	0.051
IL-33, (pg/ml)	183.97 ± 100.66	184.12 (92.30)	0.691	0.498
T1 cytokines				
IFN- γ, (pg/ml)	31.65 (11.97)	40.70 (12.44)	**<0.001**	**<0.001**
TNF-α, (pg/ml)	124.57 (111.06)	267.08 ± 93.33	**<0.001**	**<0.001**

Bold font indicates *P* < 0.05, indicating a statistically significant difference. *P* values were obtained using independent-samples *t* tests or Mann–Whitney *U* tests; *P* values (adjusted for age) were derived from analysis of covariance (ANCOVA) or GLMs.

T, type; AR, allergic rhinitis; n, number; ALOX15, arachidonate 15-lipoxygenase; CLC, Charcot–Leyden crystal; CST1, cystatin SN; ECP, eosinophil cationic protein; POSTN, periostin; TSLP, thymic stromal lymphopoietin; IL, interleukin; IFN-γ, interferon-gamma; TNF-α, tumor necrosis factor-alpha; GLMs, generalized linear models.

### 3.4 Investigation of the overall association patterns between non-classical T2 inflammatory factors and T1 cytokines, as well as the coexistence of T1 and T2 immune responses in patients with AR based on PAM-based immune clustering and subtyping and the visualization of the MCA results

PCA of age-adjusted T1 biomarkers revealed that the first PC1 accounted for 61.3% of the total variance, indicating a strong covariation between IFN-γ and TNF-α (**Figure 3A**). For T2 biomarkers, PC1 explained 29.8% of the variance, reflecting the inherent heterogeneity among the eight T2 - related markers (**Figure 3A**). Subsequently, a clustering analysis was performed using these PC1 scores as composite scores for each pathway.

**Figure 3:**
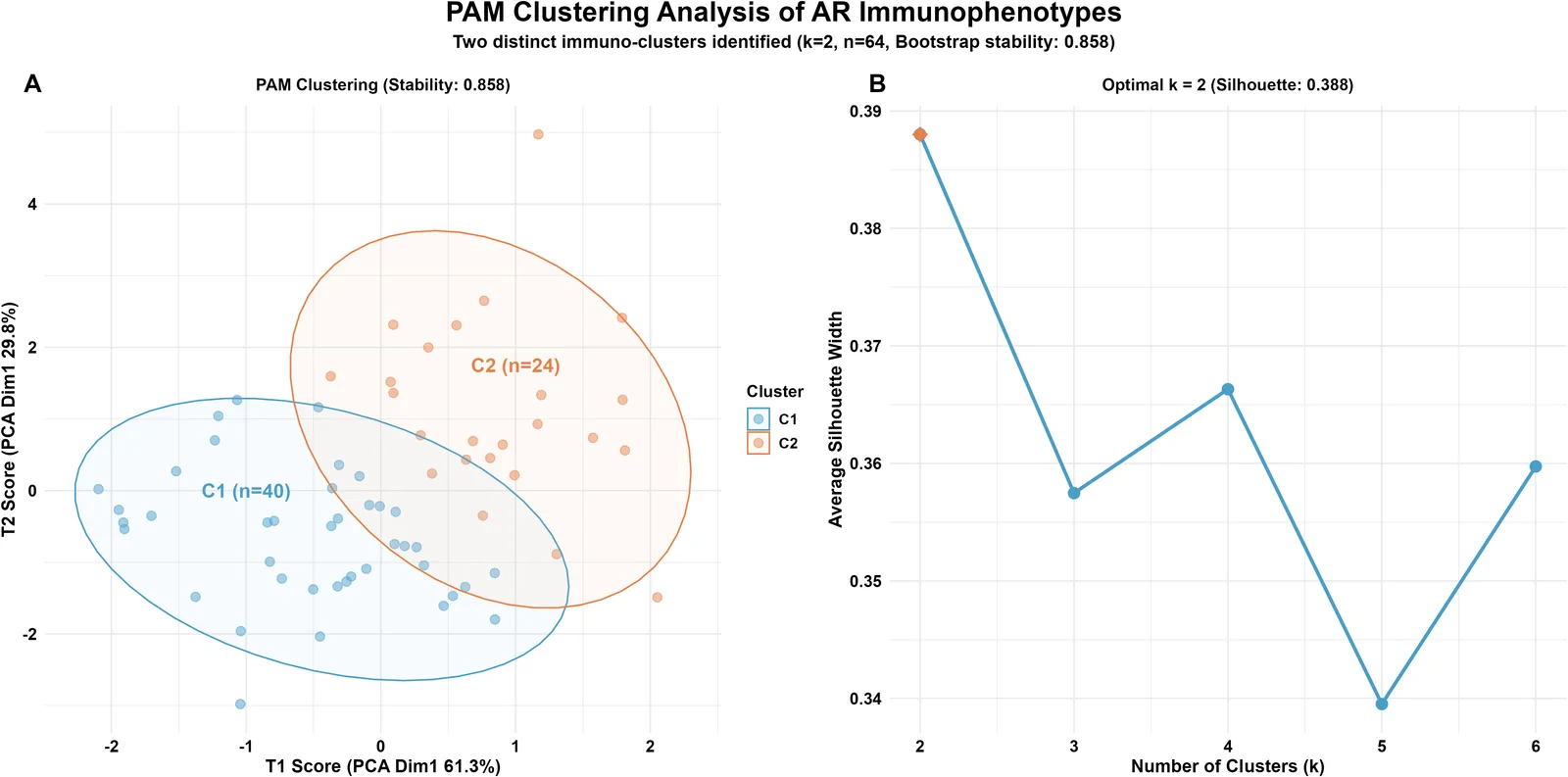
PAM clustering of immune endotypes based on PCA scores. **(A)** PCA of age-adjusted T1 and T2 biomarkers showing the variance explained by PC1 (61.3% for T1 markers and 29.8% for T2 markers) and the subsequent PAM clustering based on PC1-derived composite scores, which yielded two clusters—C1 (low T1/T2 scores, n = 40) and C2 (high T1/T2 scores, n = 24)—with a bootstrap Jaccard similarity of 0.858, confirming robust reproducibility. **(B)** The optimal number of clusters was determined as k = 2 using the PAM algorithm, with an average silhouette width of 0.388. C, cluster; PAM, partitioning around medoids; PCA, principal component analysis; T, type; PC1, principal component 1; Dim, dimension.

PAM clustering based on the PCA-derived T1 and T2 composite scores identified two optimal clusters (k = 2, average silhouette width = 0.388) (**Figure 3B**). Cluster 1 (C1, n = 40, 62.5%) was characterized by low T1 and T2 composite scores (T1: −0.52 ± 0.38; T2: −0.67 ± 0.35), whereas Cluster 2 (C2, n = 24, 37.5%) exhibited elevated scores (T1: 0.87 ± 0.48; T2: 1.11 ± 0.42) (**Figure 3A**). Based on these immune profiles, C1 was designated the low-immune-response type (LIR-AR), and C2 was designated the high-immune-response type (HIR-AR). The silhouette width of 0.388 indicated an acceptable level of cluster cohesion and separation. Furthermore, bootstrap resampling (1000 iterations) yielded an average Jaccard similarity of 0.858 ≥ 0.75 (**Figure 3A**), confirming the robust reproducibility of the cluster structure.

MCA was performed to visualize the biomarker expression patterns underlying the two clusters and identify the most discriminative biomarkers. The two dimensions of the MCA explained 36.8% of the total variance (Dim1: 21.7%, Dim2: 15.1%). The MCA variable plot (**Figure 4A**) revealed a clear separation trend between the two immunoreactive endotypes in the MCA space (Mann–Whitney *U* test, Dim1: *P* < 0.001): the centroid of C2 (HIR-AR, n = 24) was located in the positive direction of Dimension 1 (Center_Dim1 = 0.414) and spatially co-occurred closely with high-expression categories of T2-associated inflammatory factors, including CST1 (High) (Dim1 = 0.721), POSTN (High) (Dim1 = 0.695), CLC (High) (Dim1 = 0.613), and ALOX15 (High) (Dim1 = 0.416); conversely, the centroid of C1 (LIR-AR, n = 40) was located in the negative direction of Dimension 1 (Center_Dim1 = −0.248), clustering with the low-expression categories of the aforementioned inflammatory factors. These visualization results further supported the immunoreactive endotype classification pattern revealed by PAM clustering while providing an intuitive graphical basis for understanding the inflammatory molecular characteristics of each subtype. As a visualization tool, MCA yielded results consistent with the main classification conclusions of PAM clustering, with no conflicting findings.

**Figure 4:**
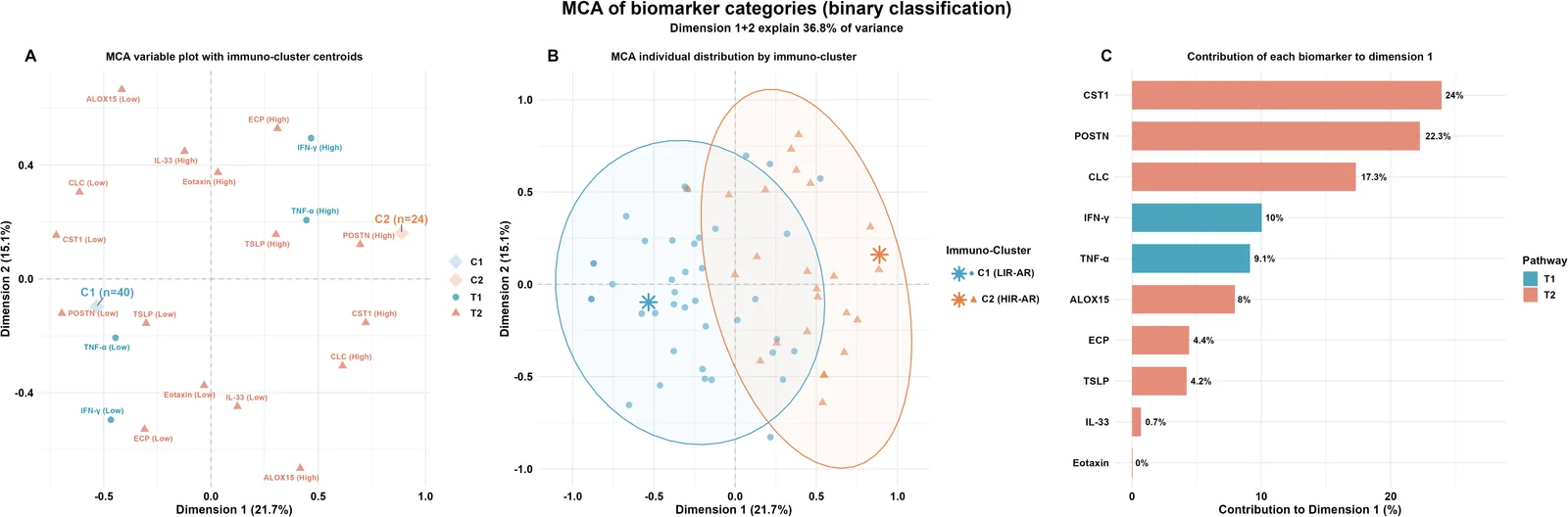
Plot of the MCA results based on the PAM clustering labels. **(A)** Spatial distribution showing C1 (LIR-AR) associated with biomarkers with low expression and C2 (HIR-AR) associated with biomarkers with high expression. **(B)** Individual distribution plot clearly demonstrating separated cluster centroids. The two dimensions of the MCA explained 36.8% of the total variance (Dimension 1: 21.7%, Dimension 2: 15.1%). **(C)** The top three discriminative variables contributing to dimension 1 were CST1 (24%), POSTN (22.3%), and CLC (17.3%). MCA, multiple correspondence analysis; PAM, partitioning around medoids; C, cluster; LIR, low-immune-response type; HIR, high-immune-response type; AR, allergic rhinitis; ALOX15, arachidonate 15-lipoxygenase; CLC, Charcot–Leyden crystal; CST1, cystatin SN; ECP, eosinophil cationic protein; POSTN, periostin; TSLP, thymic stromal lymphopoietin; IL, interleukin; IFN-γ, interferon-gamma; TNF-α, tumor necrosis factor-alpha.

Notably, IL-33 (High) was located in the negative region of Dimension 1 and distributed mainly along Dimension 2, indicating marked spatial separation from the high-expression categories of other T2 effector molecules (**Figure 4A**). This geometric pattern suggests that, in the present cohort, high IL-33 expression did not coincide with a synchronous increase in the expression of non-classical T2 effector molecules and that the two might be subject to independent regulatory mechanisms.

The plot of the distribution of individual results from MCA (**Figure 4B**) showed that the 95% confidence ellipses of the two groups partially overlapped but were predominantly separated, further suggesting that their spatial distributions were distinguishable. The contribution plot (**Figure 4C**) indicated that CST1 (24%), POSTN (22.3%), and CLC (17.3%) were the three variables contributing most to Dimension 1. These proteins may constitute a core set of T2 biomarkers distinguishing LIR-AR from HIR-AR.

### 3.5 Network analysis of T2 inflammatory factors in patients with AR

Based on the PAM clustering and MCA results, a pairwise correlation analysis of the levels of non-classical T2 factors in AR patients revealed significant positive correlations between ALOX15 and CST1 expression, CST1 and TSLP expression, CST1 and CLC expression, CLC and POSTN expression, and ECP and eotaxin expression (*P* < 0.05), while IL-33 showed no such associations. In the network, the node centrality was reflected by size and color intensity; edges denoted significant correlations (red for positive, blue for negative) (**Figure 5**). These interconnections suggest a functional module that synergistically promotes inflammation in AR patients.

**Figure 5:**
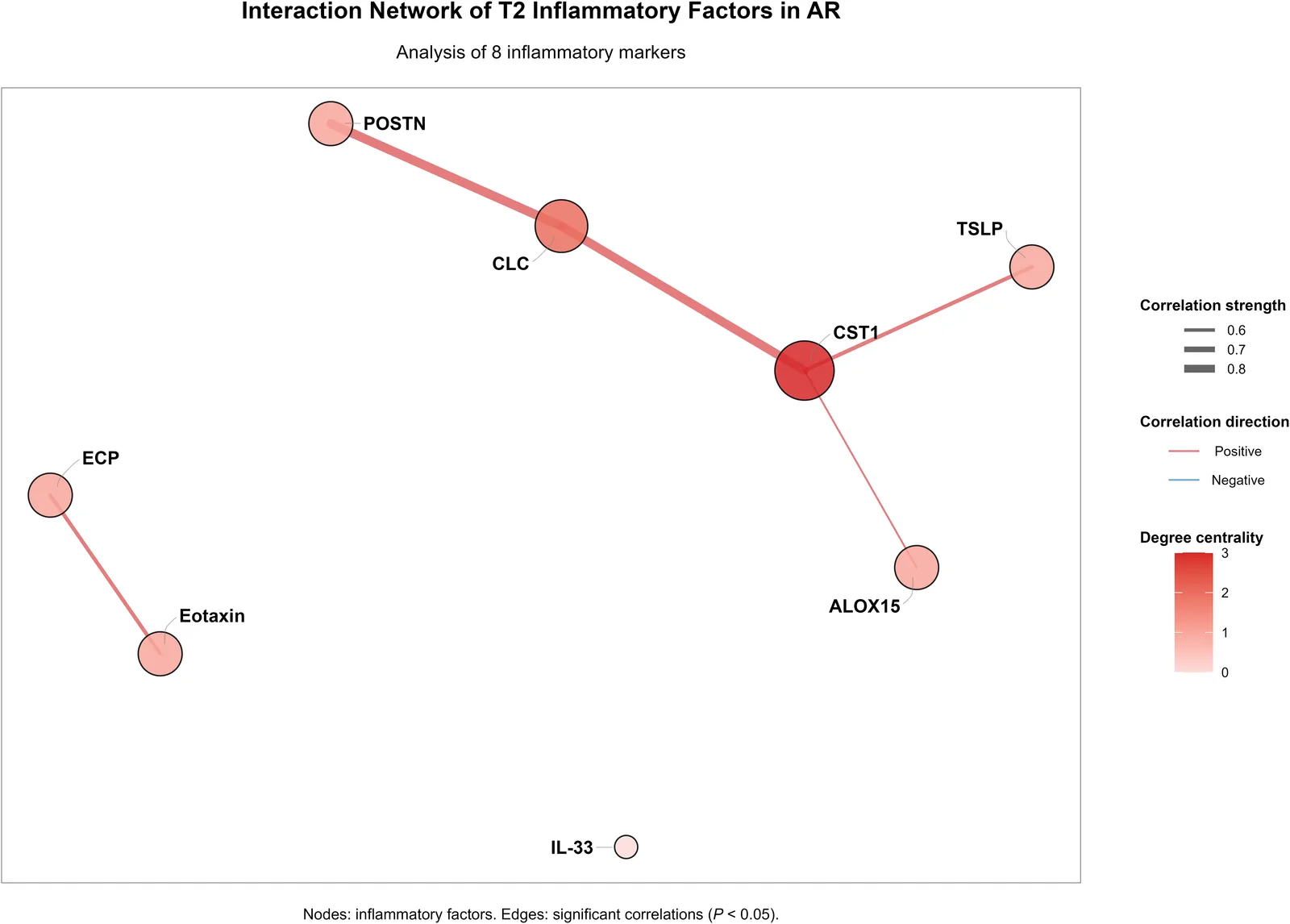
Interaction network of T2 inflammatory factors in AR. The network revealed extensive interconnectivity among the T2 inflammatory factors, whereas IL-33 showed minimal integration in the core module. Nodes in the network represented inflammatory factors; larger nodes with deeper colors reflected their position as hubs in the network. Edges (connecting lines) indicated statistically significant correlations (*P* < 0.05), with the thickness proportional to the absolute correlation coefficient and the color indicated the direction of the correlation (blue, negative; red, positive). Only significant correlations were displayed. T, type**;** AR, allergic rhinitis; IL, interleukin; ALOX15, arachidonate 15-lipoxygenase; CLC, Charcot–Leyden crystal; CST1, cystatin SN; ECP, eosinophil cationic protein; POSTN, periostin; TSLP, thymic stromal lymphopoietin.

### 3.6 Comparison of the T2/T1 ratio between AR patients and healthy controls

Based on the PAM and MCA findings, the T2/T1 inflammatory imbalance was further evaluated by calculating the ratios of the levels of non-classical T2 markers to the levels of T1 cytokines (IFN-γ and TNF-α) in AR patients and healthy controls before and after adjustment for age. After age adjustment, significantly higher ratios for most T2 markers, including ALOX15, CLC, CST1, POSTN, TSLP, and IL-33, relative to both IFN-γ and TNF-α were observed in the AR group. In contrast, ratios involving ECP and eotaxin did not differ significantly between groups (**Table 3**). These results further support the prominent roles of non-classical T2 inflammatory cytokines in AR pathogenesis.

**Table 3:** Comparison of the ratios of T2 inflammatory factors to T1 cytokines between AR patients and healthy controls.

Parameters	AR group (n = 64)	Control group (n = 42)	*P* - value	*P* - value (adjusted for age)
ALOX15/IFN- γ	26.00 (23.08)	15.42 (12.80)	**<0.001**	**<0.001**
CLC/IFN- γ	1.30 (1.06)	0.89 ± 0.29	**<0.001**	**<0.001**
CST1/IFN- γ	0.10 (0.07)	0.10 ± 0.03	0.250	**0.036**
ECP/IFN- γ	13.50 (13.06)	17.46 ± 6.38	**0.039**	0.696
Eotaxin/IFN- γ	11.32 (8.73)	15.57 ± 6.84	0.063	0.677
POSTN/IFN- γ	0.14 (0.05)	0.12 ± 0.03	**<0.001**	**<0.001**
TSLP/IFN- γ	19.39 (11.33)	16.9 ± 4.57	**0.043**	**0.013**
IL-33/IFN- γ	5.64 (4.56)	4.23 ± 1.73	**0.008**	**<0.001**
ALOX15/TNF-α	5.63 (5.32)	2.66 ± 1.30	**<0.001**	**<0.001**
CLC/TNF-α	0.27 (0.21)	0.14 ± 0.06	**<0.001**	**<0.001**
CST1/TNF-α	0.02 (0.02)	0.02 (0.01)	**<0.001**	**<0.001**
ECP/TNF-α	13.50 (13.06)	17.46 ± 6.38	**0.039**	0.696
Eotaxin/TNF-α	3.14 ± 1.73	2.50 ± 1.25	0.962	0.095
POSTN/TNF-α	0.04 ± 0.02	0.02 (0.01)	**<0.001**	**<0.001**
TSLP/TNF-α	4.79 ± 2.57	2.65 (1.06)	**<0.001**	**<0.001**
IL-33/TNF-α	1.35 (1.15)	0.65 (0.31)	**<0.001**	**<0.001**

Bold font indicates *P* < 0.05, indicating a statistically significant difference. *P* values were obtained using independent-samples *t* tests or Mann–Whitney *U* tests; *P* values (adjusted for age) were derived from analysis of covariance (ANCOVA) or GLMs.

T, type; AR, allergic rhinitis; n, number; ALOX15, arachidonate 15-lipoxygenase; IFN-γ, interferon-gamma; CLC, Charcot–Leyden crystal; CST1, cystatin SN; ECP, eosinophil cationic protein; POSTN, periostin; TSLP, thymic stromal lymphopoietin; IL, interleukin; TNF-α, tumor necrosis factor-alpha; GLMs, generalized linear models.

### 3.7 Associations of T2 inflammatory factors and T2/T1 ratios with symptom scores

The correlation analysis revealed that CST1 levels were positively correlated with rhinorrhea VAS scores (*r* = 0.254, *P* = 0.043), whereas ALOX15/IFN-γ levels were positively associated with nasal itching VAS scores (*r* = 0.271, *P* = 0.030). Negative correlations were observed between CLC levels and the total VAS score (*r* = -0.272, *P* = 0.030), as well as between the CLC/TNF-α ratio and nasal obstruction (*r* = -0.302, *P* = 0.015), sneezing (*r* = -0.247, *P* = 0.049), and total VAS scores (*r* = -0.314, *P* = 0.012). IL-33 levels (*r* = -0.336, *P* = 0.007), the IL-33/IFN-γ ratio (*r* = -0.397, *P* = 0.001), and the IL-33/TNF-α ratio (*r* = -0.322, *P* = 0.009) were all negatively correlated with SNOT-22 scores. No other indicators were significantly correlated with the VAS or SNOT-22 scores.

### 3.8 ROC curve analysis of the predictive efficacy of the ALOX15/T1 ratio for AR

Based on the stable expression of ALOX15 in both bioinformatics and serum analyses, as well as the superiority of its ratio to T1 markers, the ALOX15/T1 ratio was selected as a biomarker to evaluate its predictive diagnostic performance for AR. Both unadjusted and age-adjusted statistical models were constructed to control for the potential confounding effect of age. Prior to the age adjustment, the correlations between age and the levels of target biomarkers were assessed within the healthy control group to determine the necessity of covariate adjustment. Spearman’s correlation analyses showed that the levels of ALOX15 (*r* = 0.063, *P* = 0.692), IFN-γ *(r* = − 0.196, *P* = 0.214), and TNF-α (*r* = 0.029, *P* = 0.855) were not significantly correlated with age. These findings indicate that the age adjustment primarily served to account for demographic imbalances between the AR and control groups rather than to correct for age-related physiological variations.

We constructed age-unadjusted and age-adjusted logistic regression models, and ROC curves were generated accordingly to evaluate the predictive performance of potential biomarkers in real-world clinical practice. For the combined model, given that the ratios of these variables displayed a non-normal distribution with outliers (**Figures 6A, B**), RobustScaler was applied to its independent variables. This robust standardization was performed based on the median and IQR of the healthy control group to eliminate dimensional differences, avoid extreme regression coefficients, and further improve the stability of the combined model. The standardized variables were denoted as ()’. In the unadjusted model, the equation for the univariate model was logit (P_AR) = β_0_ + β_1_ × (ALOX15/IFN-γ or ALOX15/TNF-α), and the equation for the combined model was logit (P_AR) = β_0_ + β_1_’ × (ALOX15/IFN-γ)’ + β_2_’ × (ALOX15/TNF-α)’. The equation for the age-adjusted univariate model was logit (P_AR) = β_0_ + β_1_ × (ALOX15/IFN-γ or ALOX15/TNF-α) + β_3_ × Age. The equation for the age-adjusted combined model was logit (P_AR) = β_0_ + β_1_’ × (ALOX15/IFN-γ)’ + β_2_’ × (ALOX15/TNF-α)’ + β_3_ × Age. In these models, P_AR denotes the probability of AR onset; age was entered into the model with its original unit (years); β_0_ is the intercept term; β_1_ and β_2_ are the partial regression coefficients for raw ratio variables; β_1_’ and β_2_’ are the partial regression coefficients for standardized ratio variables; and β_3_ is the partial regression coefficient for age.

**Figure 6:**
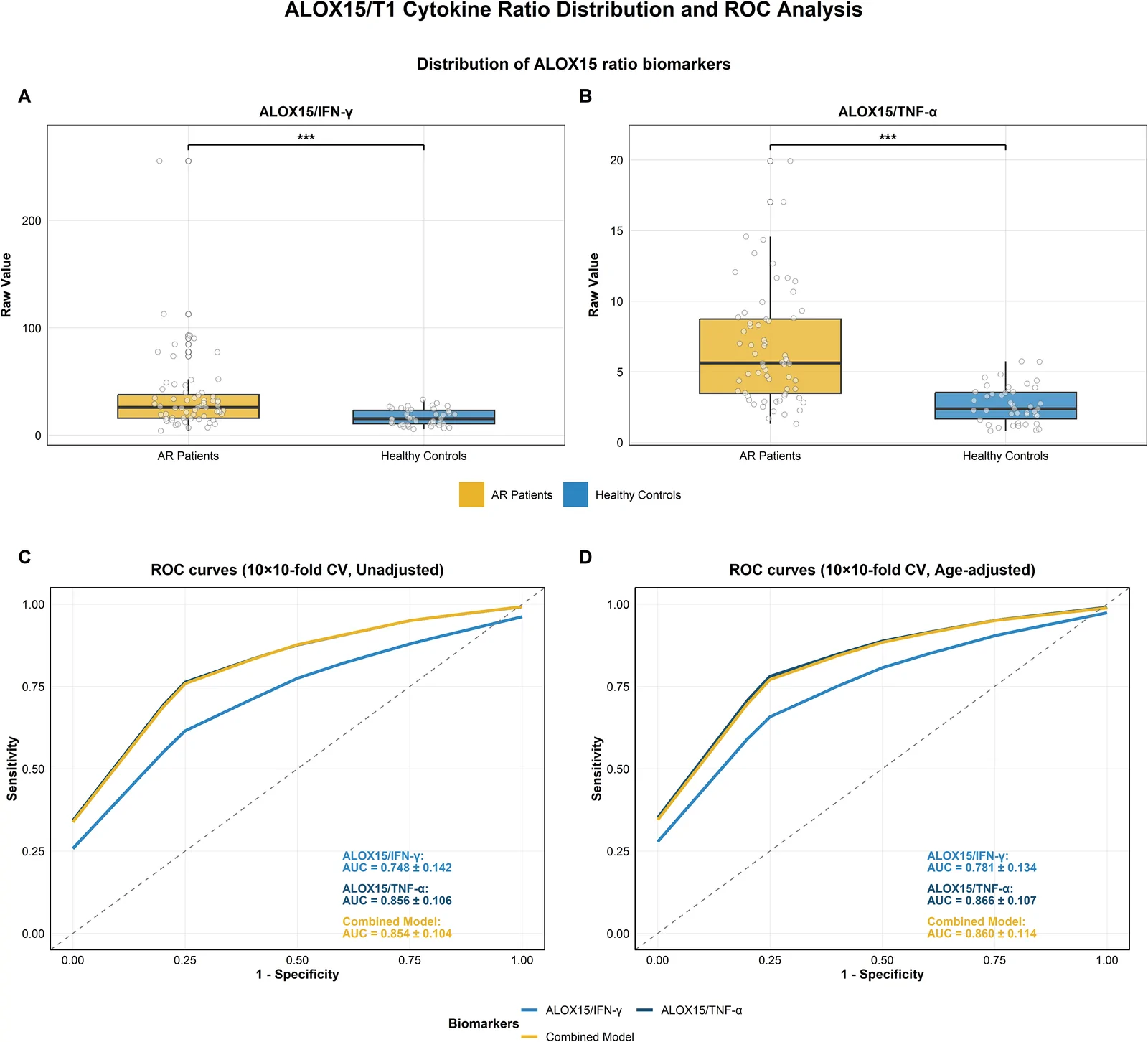
Distribution of the ALOX15/T1 cytokine ratio in the AR and control groups and the corresponding results of the ROC curve analysis. **(A, B)** Box plots showing the distribution of ALOX15/IFN-γ and ALOX15/TNF-α ratios between AR patients and healthy controls. Both ratios showed significantly higher median levels and greater variability in the AR patient group (Mann–Whitney *U* test, ^***^P < 0.001) and simultaneously exhibited a large number of high-value outliers. These characteristics provided the basis for the selection of robust normalization methods based on the median and IQR in the subsequent ROC curve analysis. **(C)** Results of the ROC curve analysis for the prediction of AR by the ratios of ALOX15 to cytokines unadjusted for age as a confounding factor: ALOX15/IFN-γ (AUC = 0.748 ± 0.142), ALOX15/TNF-α (AUC = 0.856 ± 0.106), and a combined ALOX15 ratio model (AUC = 0.854 ± 0.104). **(D)** Results of the ROC curve analysis for the prediction of AR by the ratios of ALOX15 to cytokines adjusted for age as a confounding factor: ALOX15/IFN-γ (AUC = 0.781 ± 0.134), ALOX15/TNF-α (AUC = 0.866 ± 0.107), and a combined ALOX15 ratio model (AUC = 0.860 ± 0.114). ALOX15, arachidonate 15-lipoxygenase; T, type; IFN-γ, interferon-gamma; TNF-α, tumor necrosis factor-alpha; AR, allergic rhinitis; ROC, receiver operating characteristic; AUC, area under the curve; CV, cross-validation.

Age-adjusted models incorporating age as a covariate were constructed to control for potential confounding by age. Stratified 10-fold cross-validation repeated 10 times was implemented for the internal evaluation of the predictive model using the ALOX15/IFN-γ and ALOX15/TNF-α ratios. Before the age adjustment, the ALOX15/IFN-γ ratio yielded an AUC of 0.748 ± 0.142, indicating moderate discriminative ability, whereas the ALOX15/TNF-α ratio had a higher AUC of 0.856 ± 0.106. The combined model yielded an AUC of 0.854 ± 0.104, demonstrating good discriminatory power. Notably, the age adjustment improved both the AUC values and the Youden indices of all three models, as detailed in **Figures 6C, D** and **Table 4**. No marked difference was observed between the training and validation AUCs across folds, and obvious overfitting was absent, supporting the favorable internal stability of the model (**Supplementary Table 1**).

**Table 4:** ROC curve analysis for the diagnostic value of ALOX15/T1 cytokine ratios in AR.

Variable	Age adjustment	Optimal Cut-off	Sensitivity	Specificity	Youden index	AUC (Mean ± SD)
ALOX15/IFN- γ	No	27.83	0.453	0.952	0.406	0.748 ± 0.142
ALOX15/TNF- α	No	4.364	0.672	0.905	0.577	0.856 ± 0.106
Ratio combined model	No	0.588^▲^	0.808	0.920	0.728	0.854 ± 0.104
ALOX15/IFN- γ	Yes	27.83	0.453	0.952	0.406	0.781 ± 0.134
ALOX15/TNF- α	Yes	4.364	0.672	0.905	0.577	0.866 ± 0.107
Ratio combined model	Yes	0.565^▲^	0.832	0.911	0.743	0.860 ± 0.114

▲Combined model cut-offs are based on predicted probability, whereas single biomarker cut-offs are based on raw ratios.

ROC, receiver operating characteristic; ALOX15, arachidonate 15-lipoxygenase; T, type; AR, allergic rhinitis; AUC, area under the curve; SD, standard deviation; IFN-γ, interferon-gamma; TNF-α, tumor necrosis factor-alpha.

## 4 Discussion

### 4.1 Compartmentalized expression and stratified clinical value of non-classical T2 inflammatory factors in AR patients

The results of the bioinformatics analysis revealed the significant upregulation of ALOX15, CLC, CST1 and POSTN mRNA expression in the nasal mucosa of patients with AR, indicating active local T2 inflammation. Subsequent ELISAs of serum samples showed that, after the age adjustment, only ALOX15 expression was significantly elevated in AR patients. CLC, POSTN, TSLP, and IL-33 levels were not significantly different, whereas CST1, ECP, and eotaxin levels were decreased. These discordant expression patterns may reflect differences in cellular sources, biological compartments and release mechanisms.

ALOX15 is produced by multiple immune and epithelial cells (14). ALOX15-expressing immune cells have the capacity for systemic trafficking (7), and the serum levels of this enzyme reflects the overall inflammatory burden. In contrast, CLC is regulated by local EOS activation (15); CST1 and eotaxin are mainly derived from mucosal epithelial cells (16, 17), whereas ECP is predominantly generated during bone marrow cell maturation (18). These factors constrain their release into the systemic circulation. POSTN showed inconsistent expression across the two transcriptomic datasets, and no inter-group difference was observed in serum levels, suggesting its limited value as a universal diagnostic biomarker for AR (19).These results indicate that, nasal mucosal tissue and peripheral blood represent distinct biological compartments, and their molecular expression profiles are inherently inconsistent (20); whether serum biomarkers can serve as surrogates for evaluating local inflammation is highly biomarker-specific and cannot be generalized.

This study further identified two immune clusters in AR patients based on serum-level profiles of T1/T2 biomarkers: LIR-AR (low T1/low T2) and HIR-AR (high T1/high T2). Bootstrap resampling validated the robustness of clustering (Jaccard similarity coefficient = 0.858). MCA further confirmed the biological distinctiveness of the two profiles. The co-high/co-low pattern of T1 and T2 responses indicates that they do not follow a strict “one-waxing-and-the-other-waning” relationship, but evolve dynamically in response to the microenvironment (21).

Notably, ALOX15 exhibited high consistency between transcriptomic data and serum measurements, yet it contributed only 8% to subtype discrimination in the MCA analysis. In contrast, CST1, POSTN, and CLC were highly expressed in the transcriptomic datasets, but showed limited or no significant changes at the serum protein level; collectively, these three molecules accounted for nearly 60% of the subtype-discriminating capacity. These findings suggest that biomarker values should be interpreted hierarchically based on their biological functions. ALOX15 is widely expressed and can be released into the systemic circulation (7), its serum concentration primarily reflects the systemic inflammatory burden in AR, suggesting its potential as a candidate marker for monitoring disease activity. CST1, CLC and POSTN are predominantly derived from the local nasal mucosal microenvironment and their serum expression levels are constrained by mucosal immune compartmentalization (22). Their serum alterations are more likely to reflect the systemic immune responsiveness of the body to local inflammation rather than the intensity of local inflammation itself.

Furthermore, the high expression of IL-33 did not co-occur with downstream effector molecules including ALOX15, CLC, and CST1; instead, it exhibited an opposite distribution pattern. Combined with the finding from network analysis that IL-33 showed no significant correlations with other non-classical T2 biomarkers, we hypothesize that this finding might reflect the dynamic temporal regulatory pattern during the progression of AR. As an early initiator of T2 responses (23), IL-33 induces downstream effector molecules at the early disease stage; as the inflammatory cascade intensifies, the downstream products or infiltrating immune cells may trigger a negative feedback loop, limiting the continuous high expression of IL-33. This potential mechanism of “initiation–release–negative feedback inhibition” may lead to the decoupling of serum IL-33 levels from the levels of downstream effector molecules and manifests as spatial separation in the MCA diagram.

In summary, ALOX15 may be suitable for disease monitoring, whereas CST1, POSTN and CLC may be more applicable for differential diagnosis based on immune subtyping. Collectively, they provide complementary interpretations of AR pathogenesis from two dimensions: “overall burden” and “individual responsiveness”. Nevertheless, all the above interpretations and conclusions stem from cross-sectional data and bioinformatic analyses performed on a limited sample cohort, which necessitates further validation using functional experiments and longitudinal studies.

### 4.2 Distinct clinical manifestations of AR may be linked to discrete immune-mediated mechanisms

The positive correlation between CST1 levels and rhinorrhea severity, together with previous evidence that CST1 downregulation suppressed mucus production in AR patients, suggests its potential involvement in the regulation of mucus secretion in individuals with this disease (24). Moreover, the present study revealed a positive correlation between the ALOX15/IFN-γ ratio and nasal pruritus. A previous study documented that ALOX15-catalyzed metabolites can activate transient receptor potential (TRP) channels on sensory neurons and participate in pruritus signal transduction (25). Based on these observations, we propose the following testable hypothesis: elevated levels of this ratio may (but not necessarily) be associated with a systemic immune microenvironment imbalance that favors pruritus perception. Whether ALOX15 directly mediates nasal pruritus remains to be clarified by functional experiments. Notably, all the findings of this study are derived from correlation analyses, and no causal relationships can be inferred.

In addition, negative correlations of the CLC level and the CLC/TNF-α ratio with nasal symptom scores were observed. IL-33 levels and its ratios to IFN-γ or TNF-α showed comparable associations with SNOT-22 scores. Notably, CLC serves as one of the core markers discriminating HIR-AR from LIR-AR; paradoxically, this marker, which is indicative of a high-immune-response endotype, was linked to milder symptoms. As an alarmin, increased IL-33 levels generally reflect epithelial barrier dysfunction and the onset of local T2 inflammation (23). We propose three non-mutually exclusive hypothetical interpretations to explain this apparent phenomenon of “negative correlation between inflammatory markers and symptoms”, all of which require further validation: (1) The magnitude of the immune response and symptom perception represent distinct biological dimensions. Inflammatory markers (e.g., CLC and IL-33) mirror the local (26) or systemic immune activation status, whereas the symptoms are jointly determined by downstream events, including neural reflexes, vascular permeability and glandular secretion (27). Discrepancies exist in their regulatory temporal patterns and signaling pathways. Accordingly, these markers, which serve as endotype signatures, may better reflect patients’ intrinsic immune response traits than their current symptom burden; (2) High T2 inflammation promotes immune skewing, leading to relative suppression of the TNF-α or IFN-γ pathways and consequently attenuating certain clinical symptoms driven by T1 pathways. Elevated ratios of CLC/TNF-α, IL-33/IFN-γ, or IL-33/TNF-α serve as indirect evidence of this skewing; and (3) Heterogeneous phenotypes may exist within AR (e.g., “robust inflammation–strong negative feedback” versus “robust inflammation–weak negative feedback”), in which the former is characterized by high marker expression but mild symptoms. Taken together, the negative correlations observed for CLC and IL-33 levels are more likely to reflect systemic immune skewing or compensatory regulatory processes rather than identifying symptom-specific driving pathways. Nevertheless, due to the cross-sectional study design, the aforementioned interpretations remain exploratory hypotheses and await validation via functional assays and longitudinal investigations.

Of note, the level of IL-8, a potent chemokine produced by neutrophils, is markedly elevated in both the nasal mucosa and serum of patients with AR. The inflammatory network involving IL-8 may transcend the canonical T1/T2 immune classification framework, further indicating that a single marker or a small panel of markers cannot fully decipher the complexity of symptom-relevant immune pathways (28). Future research should focus on interaction networks among multiple inflammatory mediators to comprehensively elucidate the immunological mechanisms of symptom manifestation in AR patients.

### 4.3 The T2/T1 ratio may be used as an effective predictor of AR

Based on the stable expression of ALOX15 and its role in regulating the T1/T2 balance, univariate models were developed using the ALOX15/IFN-γ and ALOX15/TNF-α ratios to predict AR. Since age has been reported as a potential confounding factor for inflammatory marker levels (29), it was further adjusted in the analysis. Notably, the AUC values for ALOX15-derived ratios exhibited a modest increasing trend upon adjustment for age. Although age could act as a potential confounding variable, the ALOX15/TNF-α ratio still appeared to yield acceptable predictive performance, with an AUC of 0.856 ± 0.106.

We objectively evaluated the potential value of this ratio by comparing its diagnostic performance with that of previously reported AR biomarkers. Previous studies have demonstrated that the levels of TGF-β1 (AUC = 0.928, sensitivity = 0.836, specificity = 0.860), IL-13 (AUC = 0.922, sensitivity = 0.803, specificity = 0.901), and serum IgE (AUC = 0.922, sensitivity = 0.852, specificity = 0.939) exhibit prominent diagnostic utility for AR (30). Numerically, the ALOX15/TNF-α ratio (AUC = 0.856) yielded lower values than these reported biomarkers. Nevertheless, the major strength of this ratio lies in the integration of the ALOX15-governed lipid metabolic pathway and the TNF-α-driven inflammatory pathway within a unified framework, which may partially mirror the T1/T2 immune imbalance implicated in AR pathogenesis. However, the sensitivity of this ratio remains limited, and its relatively high specificity is largely cutoff-dependent. Moreover, because these comparisons derive from distinct study populations, direct head-to-head validation within a single cohort is required to benchmark its diagnostic performance against established biomarkers such as serum IgE. Thus, while the ALOX15/TNF-α ratio may offer complementary insights into T1/T2 immune dysregulation, further evidence—including prospective studies with external validation—is needed to confirm its clinical utility and to determine whether it can be integrated into the current diagnostic algorithm for AR.

### 4.4 Limitations of this study and future research directions

In this study, systematic analyses of biomarker screens, immunotyping, and diagnostic models were conducted, and the reliability of the results was confirmed through multiple internal validation strategies, including LOOCV, bootstrap resampling, and 10-fold cross-validation. Nevertheless, from a methodological perspective, several limitations remain to be addressed in future research.

Study limitations: First, the initial discovery dataset (GSE43523) has a limited sample size (7 AR patients vs. 5 healthy controls). Although internal validation confirmed the stability of the candidate genes and the results from GSE101720 (17 AR patients vs. 9 healthy controls) replicated the key trends, large-scale initial screening and validation of DEGs were not feasible due to severe batch effects, data heterogeneity, and a lack of suitable datasets for integration. Therefore, the current findings should be interpreted as exploratory. Second, the discordant expression of non-classical T2 inflammatory factors (e.g., CLC and CST1) between nasal mucosal tissue and serum lacks direct supportive evidence from tissue-level protein validation or cell-specific functional assays. Although we have proposed possible explanations such as “cellular sources” and “mucosal immune compartmentalization”, these inferences remain speculative and await further experimental verification. Third, this study is a single-center cross-sectional study (n = 64) and cannot assess the dynamic changes in immune subtypes or biomarkers over time or in response to treatment, and thus the generalizability of the findings to broader populations remains to be established. Fourth, although the ALOX15/TNF-α composite ratio model displayed acceptable internal stability through cross-validation, its sensitivity remained modest, and its specificity was largely dependent on the chosen cutoff value. Furthermore, external validation and head-to-head comparisons with established biomarkers are lacking for this ratio. Therefore, no definitive conclusion regarding its diagnostic utility can be drawn at this stage.

Future research directions: First, systematic external validation of the candidate biomarkers (e.g., ALOX15 and CST1), PAM-based immunotyping, and the diagnostic performance of the composite ratio model should be conducted in independent, multicenter, large-sample cohorts using standardized assay protocols. Second, tissue-level protein validation and cell-specific analyses (e.g., immunohistochemistry, immunofluorescence staining, or single-cell RNA sequencing) are warranted to elucidate the mechanisms underlying the discordant expression patterns between the transcriptomic and serum protein levels of the aforementioned non-classical T2 inflammatory factors. Third, longitudinal follow-up designs should be adopted to evaluate the dynamic changes in subtypes and biomarkers in response to treatment. Additionally, functional studies on ALOX15 are needed to clarify its pathophysiological role in AR pathogenesis. Fourth, head-to-head comparisons with established biomarkers (e.g., serum IgE levels) along with prospective external validation are needed to determine whether the ALOX15/TNF-α ratio can serve as an auxiliary indicator for an AR diagnosis.

In summary, the findings of the present study are exploratory. We anticipate that rigorously designed external validation and functional experiments in future studies will provide more robust scientific evidence to support clinical translation.

## 5 Conclusions

By integrating bioinformatics and clinical validation, this study characterized the potential role of non-classical T2 inflammatory factors, such as ALOX15, in AR, including lipid metabolism, inflammation, eosinophilic inflammation, epithelial barrier stress and tissue remodeling, which together may contribute to the pathophysiology of AR. The consistent expression of ALOX15 between nasal mucosal tissue and serum supports its potential utility as a biomarker for disease monitoring in patients with AR. Based on the serum T1/T2 immune profiles, AR patients were classified into two subtypes in this study: LIR-AR and HIR-AR. CST1, POSTN, and CLC were identified as the core discriminatory variables distinguishing the two subtypes and may hold promise for immunotype differentiation. The different clinical symptoms of AR patients may be related to different immune mechanisms. The ALOX15/TNF-α ratio may serve as a useful additional indicator for the diagnosis of AR. However, as the above symptom-correlation findings are derived from statistical correlation analyses, causal relationships cannot be inferred. These conclusions remain exploratory and warrant further validation in larger independent cohorts and through functional studies.

## Data Availability

The raw data supporting the conclusions of this article will be made available by the authors, without undue reservation.

## Supplementary material

Supplementary Figure 1 Assessment of the stability of the DEGs in the GSE43523 dataset using LOOCV. The LOOCV analysis demonstrated that the changes in the expression of core DEGs, including ALOX15, CST1, and CLC, remained robust across all iterations in AR patients. The x-axis represents the log2FC values derived from the original dataset, while the y-axis represents the average log2FC values after the LOOCV. The dashed line indicates the line of perfect concordance (y = x). The blue circles denote core DEGs that exhibited stable performance across cross-validation rounds; the red circles represent low-abundance or unstable genes that failed to maintain consistent expression changes. DEGs, differentially expressed genes; LOOCV, leave-one-out cross-validation; ALOX15, arachidonate 15-lipoxygenase; CST1, cystatin SN; CLC, Charcot–Leyden crystal; AR, allergic rhinitis; log2FC, log2 fold change.

Supplementary Figure 2 Bootstrap resampling analysis of the stability of upregulated DEGs in GSE43523. Bootstrap resampling (n = 1000 iterations) further confirmed the robustness of the significantly upregulated genes. For each gene, the 95% confidence intervals of the log2FC values are shown. Genes with confidence intervals that did not cross zero (i.e., the lower bound > 0) were considered statistically stable and reliable. ALOX15, CLC, and CST1 all had 95% confidence intervals excluding zero, supporting their stable upregulation in AR patients. The blue circles denote genes with stable expression, whereas the red circles denote genes with unstable expression or low abundance. DEGs, differentially expressed genes; log2FC, log2 fold change; ALOX15, arachidonate 15-lipoxygenase; CLC, Charcot–Leyden crystal; CST1, cystatin SN; ECP, eosinophil cationic protein; POSTN, periostin; TSLP, thymic stromal lymphopoietin; IL, interleukin; IFN-γ, interferon-gamma; TNF-α, tumor necrosis factor-alpha; AR, allergic rhinitis.

## References

[1] Zhang Y Lan F Zhang L . Advances and highlights in allergic rhinitis. Allergy. (2021) 76:3383–9. doi: 10.1111/all.15044 34379805

[2] Wise SK Damask C Roland LT Ebert C Levy JM Lin S . International consensus statement on allergy and rhinology: Allergic rhinitis – 2023. Int Forum Allergy Rhinol. (2023) 13:293–859. doi: 10.1002/alr.23090 36878860

[3] Kim CK Hwang Y Song DJ Yu J Sohn MH Park YM . Efficacy and safety of montelukast+levocetirizine combination therapy compared to montelukast monotherapy for allergic rhinitis in children. Allergy Asthma Immunol Res. (2024) 16:652–67. doi: 10.4168/aair.2024.16.6.652 39622689 PMC11621474

[4] Bernstein JA Bernstein JS Makol R . Allergic rhinitis: A review. JAMA. (2024) 331:866–77. doi: 10.1001/jama.2024.0530 38470381

[5] Zhang P . The role of diet and nutrition in allergic diseases. Nutrients. (2023) 15:3683. doi: 10.3390/nu15173683 37686715 PMC10490368

[6] Yu X Wang M Cao Z . Reduced CD4+T cell CXCR3 expression in patients with allergic rhinitis. Front Immunol. (2020) 11:581180. doi: 10.3389/fimmu.2020.581180 33224143 PMC7669911

[7] Xu X Li J Zhang Y Zhang L . Arachidonic acid 15-lipoxygenase: Effects of its expression, metabolites, and genetic and epigenetic variations on airway inflammation. Allergy Asthma Immunol Res. (2021) 13:684–96. doi: 10.4168/aair.2021.13.5.684 34486255 PMC8419644

[8] Qin R Long F Zhang P Huang R Hu H Guo Y . Presence of sputum IgG against eosinophilic inflammatory proteins in asthma. Front Immunol. (2024) 15:1423764. doi: 10.3389/fimmu.2024.1423764 39091502 PMC11291201

[9] Ishida A Ohta N Suzuki Y Kakehata S Okubo K Ikeda H . Expression of pendrin and periostin in allergic rhinitis and chronic rhinosinusitis. Allergol Int. (2012) 61:589–95. doi: 10.2332/allergolint.11-OA-0370 22918213

[10] Wang M Gong L Luo Y He S Zhang X Xie X . Transcriptomic analysis of asthma and allergic rhinitis reveals CST1 as a biomarker of unified airways. Front Immunol. (2023) 14:1048195. doi: 10.3389/fimmu.2023.1048195 36733482 PMC9888248

[11] Zoabi Y Levi-Schaffer F Eliashar R . Allergic rhinitis: Pathophysiology and treatment focusing on mast cells. Biomedicines. (2022) 10:2486. doi: 10.3390/biomedicines10102486 PMC9599528 36289748

[12] Bousquet J Hellings PW Agache I Amat F Annesi-Maesano I Ansotegui IJ . Allergic rhinitis and its impact on asthma (ARIA) phase 4 (2018): Change management in allergic rhinitis and asthma multimorbidity using mobile technology. J Allergy Clin Immunol. (2019) 143:864–79. doi: 10.1016/j.jaci.2018.08.049 30273709

[13] Gu J Qin G Jiang L Xu W Wang Y Liao J . Correlations between IL-36 family cytokines in peripheral blood and subjective and objective assessment results in patients with allergic rhinitis. Allergy Asthma Clin Immunol. (2023) 19:79. doi: 10.1186/s13223-023-00834-y 37649097 PMC10470177

[14] Ito E Hayashizaki R Hosaka T Yamane T Miyata J Isobe Y . Eosinophils and pleural macrophages counter regulate IL-33-elicited airway inflammation via the 12/15-lipoxygenase pathway. Front Immunol. (2025) 16:1565670. doi: 10.3389/fimmu.2025.1565670 40313934 PMC12043891

[15] Gevaert E Delemarre T De Volder J Zhang N Holtappels G De Ruyck N . Charcot-Leyden crystals promote neutrophilic inflammation in patients with nasal polyposis. J Allergy Clin Immunol. (2020) 145:427–30. doi: 10.1016/j.jaci.2019.08.027 31493418

[16] Moon S Moon S Lee S Kim G Koo MS Cho HJ . Epithelial lining fluid cystatin SN is a noninvasive biomarker for predicting type 2 chronic rhinosinusitis. Allergy. (2025) 80:2572–85. doi: 10.1111/all.16652 40689541 PMC12444822

[17] Kang Z Xu L Ai P Qu W Li T Wang L . Expression and correlation of SOCS3 and Eotaxin mRNA and proteins levels in nasal mucosal tissue of allergic rhinitis patients. Front Immunol. (2025) 16:1561650. doi: 10.3389/fimmu.2025.1561650 40568590 PMC12187767

[18] Gerstberger S Hafner M Tuschl T . A census of human RNA-binding proteins. Nat Rev Genet. (2014) 15:829–45. doi: 10.1038/nrg3813 25365966 PMC11148870

[19] Wang H Zhang Q Hu M Shao J Wang G . Periostin in allergic rhinitis: From pathogenic mediator to predictive biomarker and therapeutic target. Front Pharmacol. (2026) 17:1801272. doi: 10.3389/fphar.2026.1801272 42394977 PMC13322882

[20] Watts AM West NP Smith PK Cripps AW Cox AJ . Adult allergic rhinitis sufferers have unique nasal mucosal and peripheral blood immune gene expression profiles: A case-control study. Immun Inflammation Dis. (2022) 10:78–92. doi: 10.1002/iid3.545 34637606 PMC8669689

[21] Zhu Y Yu J Zhu X Yuan J Dai M Bao Y . Experimental observation of the effect of immunotherapy on CD4+ T cells and Th1/Th2 cytokines in mice with allergic rhinitis. Sci Rep. (2023) 13:5273. doi: 10.1038/s41598-023-32507-6 37002325 PMC10066377

[22] Zhang Z Zhang X Li J Song Y Xi L Wang C . Local IgE is a superior biomarker than serum IgE for nasal type 2 inflammation in house dust mites sensitized allergic rhinitis. Rhinology. (2026) 64:113–21. doi: 10.4193/Rhin25.381 41171089

[23] Duchesne M Okoye I Lacy P . Epithelial cell alarmin cytokines: Frontline mediators of the asthma inflammatory response. Front Immunol. (2022) 13:975914. doi: 10.3389/fimmu.2022.975914 36311787 PMC9616080

[24] Gao W Jin Z Zheng Y Xu Y . Psoralen inhibits the inflammatory response and mucus production in allergic rhinitis by inhibiting the activator protein 1 pathway and the downstream expression of cystatin-SN. Mol Med Rep. (2021) 24:652. doi: 10.3892/mmr.2021.12291 34278468 PMC8299190

[25] Yoo S Lim JY Hwang SW . Sensory TRP channel interactions with endogenous lipids and their biological outcomes. Molecules. (2014) 19:4708–44. doi: 10.3390/molecules19044708 24739932 PMC6271031

[26] Nyenhuis SM Alumkal P Du J Maybruck BT Vinicky M Ackerman SJ . Charcot-Leyden crystal protein/galectin-10 is a surrogate biomarker of eosinophilic airway inflammation in asthma. biomark Med. (2019) 13:715–24. doi: 10.2217/bmm-2018-0280 31157540 PMC6630493

[27] Sharma A Deshmukh P Jain S Gaurkar S Sharma A . Unraveling the molecular threads: A comprehensive review of the pathogenesis and therapeutic insights into allergic rhinitis. Cureus. (2024) 16:e64410. doi: 10.7759/cureus.64410 39131035 PMC11317112

[28] Cergan R Berghi O Dumitru M Vrinceanu D Manole F Musat GC . Interleukin 8 molecular interplay in allergic rhinitis and chronic rhinosinusitis with nasal polyps: A scoping review. Life (Basel). (2025) 15:469. doi: 10.3390/life15030469 40141813 PMC11943928

[29] Boots EA Feinstein DL Leurgans S Aiken-Morgan AT Fleischman DA Lamar M . Acute versus chronic inflammatory markers and cognition in older black adults: Results from the Minority Aging Research Study. Brain Behav Immun. (2022) 103:163–70. doi: 10.1016/j.bbi.2022.04.014 35439553 PMC9704497

[30] Liu Y Kong Y Zhou X . Screening and analysis for potential clinical diagnostic and prognostic markers in allergic rhinitis. Am J Transl Res. (2024) 16:2670–82. doi: 10.62347/GKZE5945 39006280 PMC11236636

