# Correction: Identifying immune burden status and predictors via the non-classical type 2 inflammatory axis in allergic rhinitis

**DOI:** 10.3389/fimmu.2026.2016752

**Published:** 2026-10-08

**Authors:** Yi He, Beirong Liu, Xuegui Liu, Xinman Hu, Qing Liu, Zhuoping Liang

**Affiliations:** Department of Otolaryngology Head and Neck Surgery, the Affiliated Hospital of Southwest Medical University, Luzhou, China

**Keywords:** allergic rhinitis, biomarker, clinical response, immunity, local immune signature

There was a mistake in **Table 1** as published. The published **Table 1** incorrectly displays the table intended for **Supplementary Material** due to an upload mix‐up; this error does not affect the results or conclusions of the paper. The corrected **Table 1** appears below.

**Table 1:** Comparison of baseline characteristics between subjects in the AR group and the control group.

Parameters	AR group (n=64)	Control group (n=42)	*P* - value
Gender			
Male, n (%)	35 (54.69)	15 (35.71)	0.056
Female, n (%)	29 (45.31)	27 (64.29)	
Age	18 (19.00)	26 (11.00)	**0.005**
Asthma, n (%)	2 (3.13)	0 (0.00)	0.517
Smoking, n (%)	1 (1.56)	2 (4.76)	0.561
Nasal symptom VAS score			
Obstruction score	5.61 ± 2.67	N/A	–
Rhinorrhea score	6.00 (4.00)	N/A	–
Sneezing score	6.00 (4.00)	N/A	–
Nasal itching score	5.41 ± 2.82	N/A	–
Total nasal symptom score	22.89 ± 9.36	N/A	–
SNOT-22 score	27.50 (22.71)	N/A	–

Bold font indicates *P* < 0.05, indicating a statistically significant difference. AR, allergic rhinitis; n, number; VAS, visual analog scale; N/A, not applicable; SNOT-22, twenty-two-item sino-nasal outcome test.

The original version of this article has been updated.

