# Supplementary material for: Identifying immune burden status and predictors via the non-classical type 2 inflammatory axis in allergic rhinitis
Source: Front Immunol. 2026 Sep 30;17:1936486. doi: 10.3389/fimmu.2026.1936486 (PMC13689755; doi:10.3389/fimmu.2026.1936486)
Supplement: Supplementary file 1 [file Table1.docx]

Supplementary Material

Supplementary TABLE 1 Assessment of the overfitting of the diagnostic models

| Model | Type | Train_AUC | Cross-validated _AUC | Cross-validated _SD | Difference | Status |
| --- | --- | --- | --- | --- | --- | --- |
| ALOX15/IFN-γ | Unadjusted | 0.749 | 0.748 | 0.142 | 0.001 | No overfitting |
| ALOX15/IFN-γ | Age-adjusted | 0.787 | 0.781 | 0.134 | 0.006 | No overfitting |
| ALOX15/TNF-α | Unadjusted | 0.856 | 0.856 | 0.106 | 0.000 | No overfitting |
| ALOX15/TNF-α | Age-adjusted | 0.874 | 0.866 | 0.107 | 0.008 | No overfitting |
| Combined Model | Unadjusted | 0.858 | 0.854 | 0.104 | 0.004 | No overfitting |
| Combined Model | Age-adjusted | 0.874 | 0.860 | 0.114 | 0.014 | No overfitting |

The AUC for the training set was calculated using the full dataset (n = 106); the cross-validated AUC was presented as the mean ± SD from 10-fold cross-validation repeated 10 times. Difference = Training AUC − Cross-validated AUC. An absolute difference < 0.05 indicates no overfitting.

AUC, area under the curve; SD, standard deviation; ALOX15, arachidonate 15-lipoxygenase; IFN-γ, interferon-gamma; TNF-α, tumor necrosis factor-alpha.
